# Vicarious value learning by differential outcomes training: A social transfer of control methodology

**DOI:** 10.1016/j.mex.2021.101294

**Published:** 2021-03-02

**Authors:** Robert Lowe, Jonathan Rittmo, Rickard Carlsson, Pierre Gander

**Affiliations:** a Department of Applied Information Technology, University of Gothenburg, Gothenburg, Sweden; b School of Philosophy, Psychology and Language Sciences, University of Edinburgh, Edinburgh, UK

**Keywords:** Social learning, Emotion processing, Inference, Memory

## Abstract

This article adapts an existing experimental protocol for assessing individuals’ ability to transfer knowledge across instrumental and pavlovian learning stages. The protocol (*Transfer of Control* using differential outcomes learning) is adapted to fit social contexts wherein the pavlovian learning phase is modulated so that individuals are able to observe, and potentially learn from, the stimulus associated with reinforcing outcomes presented to another (observable) individual. Transfer of Control concerns participants combining knowledge of learned instrumental and pavlovian (stimulus, response, outcome) associations in order to ground the learning of new associations. The article describes the theoretical and procedural underpinnings of a novel *Social Transfer of Control* methodology. The use of such a methodology is two-fold: i) to serve as a guide to researchers interested in evaluating how individuals can learn from others in a partially observable setting, i.e. when behavioural and reinforcing outcome information is hidden, and bring to bear this knowledge on their own instrumental decision making; ii), to facilitate investigation of the routes of cognitive and emotional empathy, with potential applications for educational and clinical settings.

• Three stage *Transfer of Control* behavioural methodology is adapted to include a social (pavlovian) learning stage.
• Hypotheses can be tested that concern learning rewarding instrumental responses achieved by observation of others’ emotionally expressive reactions to differentially rewarding outcomes.
• Methodological and validation considerations for evaluating the above are presented

## Graphical abstract

**Figure:**
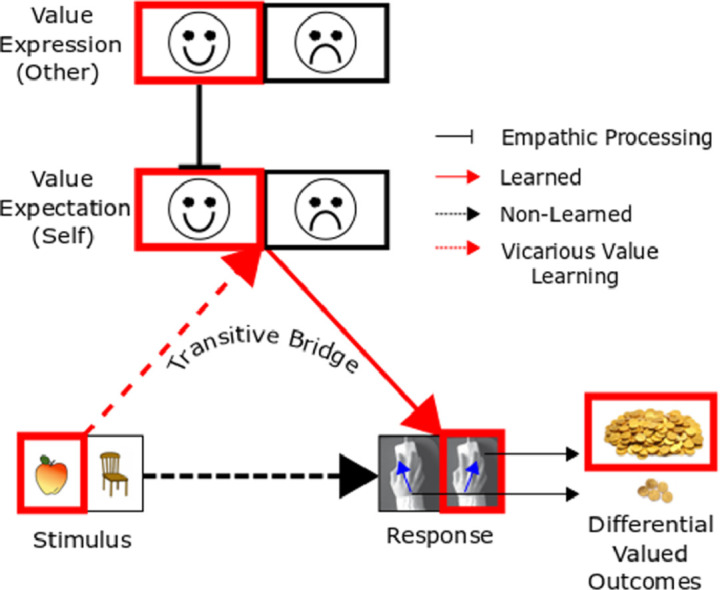

Specifications Table

**Table:** 

Subject Area:	Psychology
More specific subject area:	Behavioural Psychology
Method name:	Differential Outcomes Social Transfer of Control
Name and reference of original method:	We provide an initial theoretical justification for the approach in: R. Lowe, A. Almér, G. Lindblad, P. Gander, P., J. Michael, C. Vesper, Minimalist social-affective value for use in joint action: A neuralcomputational hypothesis. *Frontiers in Computational Neuroscience*, 10 (2016) 88. We provide our first experimental validation of the approach in: J. Rittmo, R. Carlsson, P. Gander, R. Lowe, Vicarious value learning: Knowledge transfer through affective processing on a social differential outcomes task, *Acta Psychologica*. https://doi.org/10.1016/j.actpsy.2020.103134
Resource availability:	Raw data from the above referenced experimental work can be found in the accompanying article: Gander, P., Rittmo, J., Carlsson, R., & Lowe, R. (2020). A social differential outcomes learning task: Performance, EEG, and questionnaire data. *Data in Brief*, 33, 106590.

*Method details

## Differential outcomes and Transfer of Control Protocols: Theory-based Methodological Considerations

**Fig. 1:**
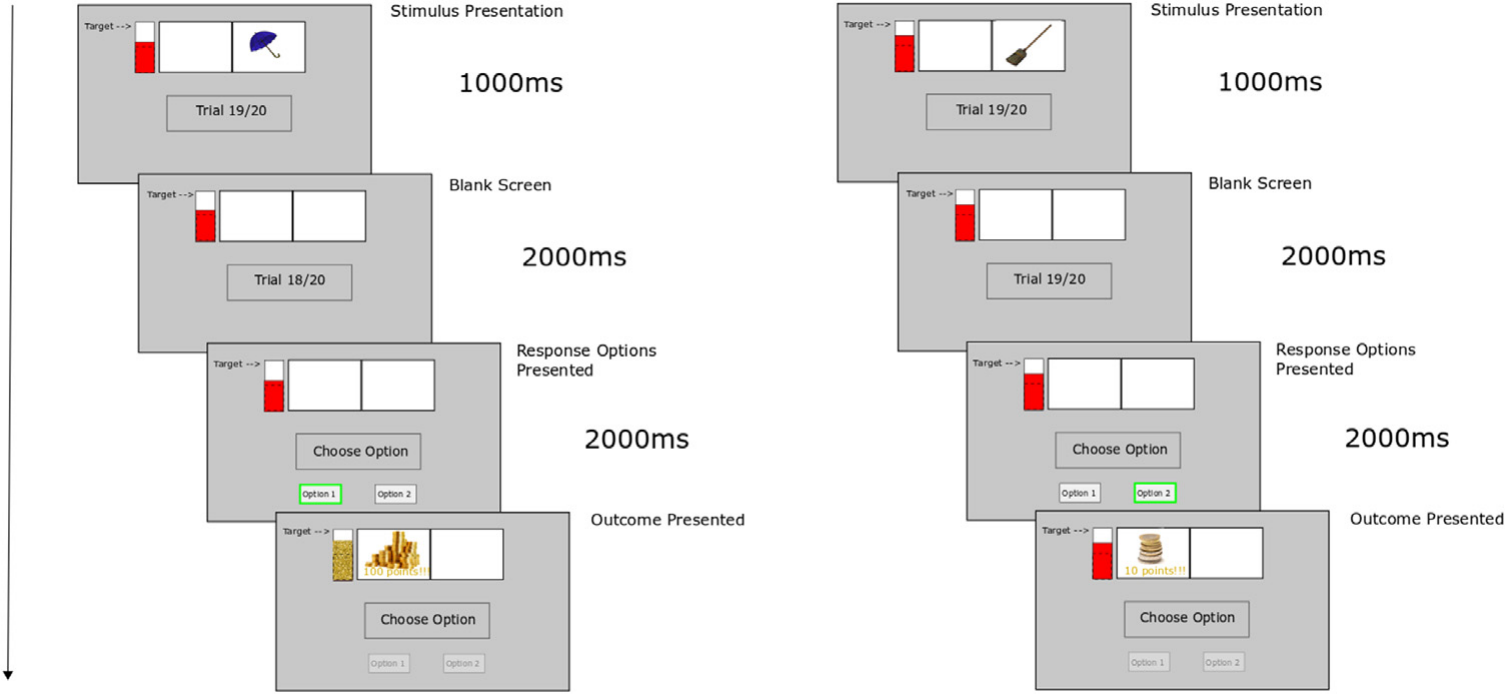
Example Trial Progression used in a Different Outcomes Training Set-Up (adapted from [4]). Left. Example trial progression. A stimulus (umbrella image taken from [5] standardized dataset) is presented (top image) for a short period, then withdrawn. Two response options (button tabs) are made available – green boxed represents chosen response – this was not highlighted during the task). Responses are greyed out once a choice is made. The rewarding (high or low) outcome is then presented (alternatively a “punishment” red circle with line through is shown for incorrect response). Right. Example trial progression for alternative S-R-O pairing. A different stimulus (brush) – response (option 2) pairing leads to a different (low scoring) reward. Differential rewarding outcome visual presentations were accompanied with differential sounds: sound of a slot machine depositing a lot of coins/tokens for high reward, sound of a slot machine “chuh-ching” but without large coin/token deposit sound for low reward.

In this sub-section we consider *theory-based* methodological considerations for designing a human-participant social transfer of control experiment. Differential outcomes training (DOT) is a well-studied procedure [1,2] for evaluating the learning capabilities of humans and non-human animals on memory and decision-making tasks where (rewarding) outcomes differ according to the trial-specific preceding cue stimulus. The procedure concerns presenting to participants, on each trial, i) an arbitrary stimulus (S), e.g. in a computerized task, an image of a brush, shortly followed by ii) two or more behavioural response (R) options, e.g. tab buttons on the left and right of the screen, for which the ‘correct' response (learned through trial and error) produces iii) an outcome (O), e.g. image of cash reward. For an example of this S-R-O sequence see Fig. 1 (and https://robertlowe2.gitlab.io/publii-dicelabbers/epi-project.html for video example). In contrast to classical behavioural experiments where the same reinforcing outcome is presented irrespective of the particular correct stimulus cued responses, DOT entails presentation of rewarding (or at least non-negative) outcomes specific (differential) to a given stimulus-response pair (Fig. 1).

Standardly, the differential outcomes training procedure entails a single stage of training over a number of learning trials where the objective is for participants to learn to associate different stimuli with different responses following repeated alternating presentations of *each* of the stimuli and *all* of the response options. The use of differential rewarding outcomes (for each correct stimulus-response pair) has been robustly found to speed up learning as compared to a non-differential (or common) outcomes control [3]. This is known as the *differential outcomes effect*.

**Fig. 2:**
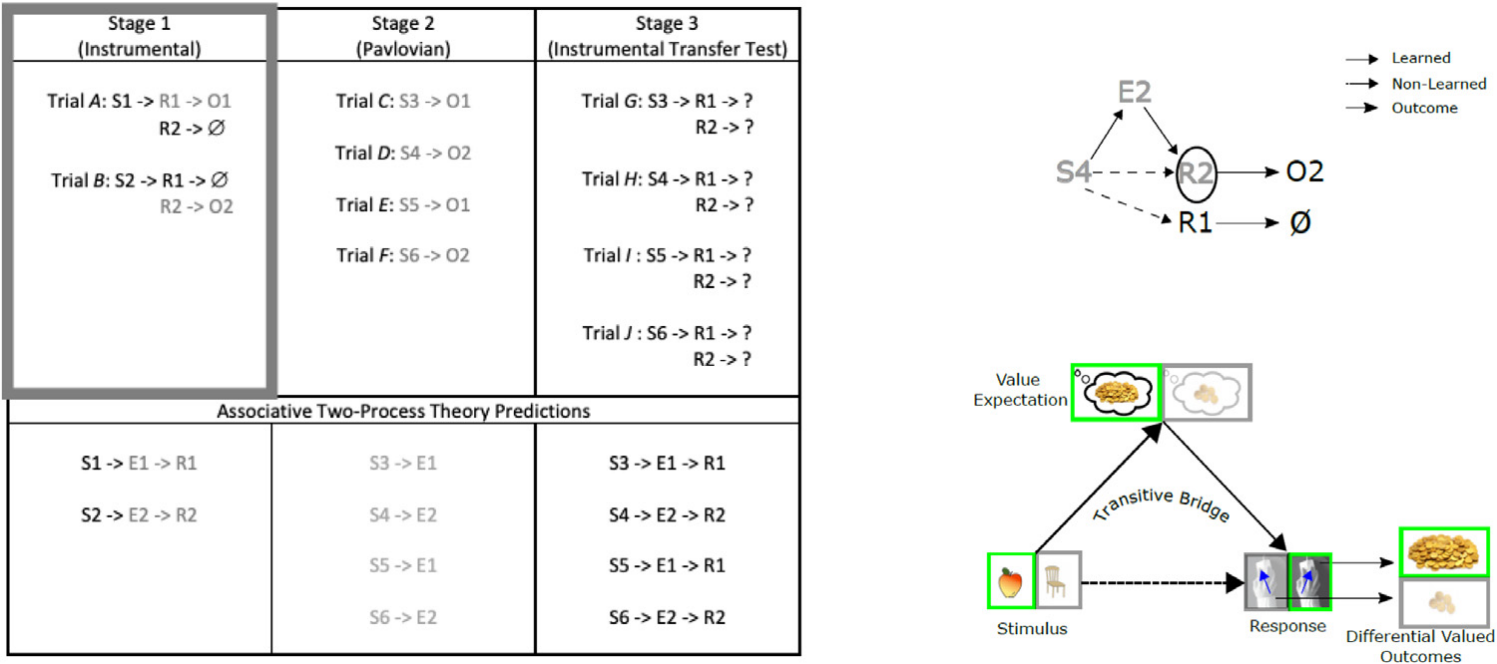
Transfer of Control (ToC) Protocol (adapted from [4]). Left. Example three-stage ToC where participants learn stimulus-response (S-R) and stimulus-outcome (S-E) associations in Instrumental and Pavlovian stages. The participants are tested (Stage 3) to see whether they are able to transfer knowledge (E-R, Stage 1; S-E, Stage 2) to bring to bear on response selection in Stage 3. In the example, according to Associative Two-Process theory, by Stage 3, participants in the first trials, i.e. without learning, should show a selection bias towards R2 when presented with either S4 or S6 due to learning in the previous two stages and for R1 when presented with either S3 or S5. This is the case in spite of never having learned these S-R pairings in Stage 1 or Stage 2. Right. Associative Two-Process depiction of the associative transitive inferential ‘bridge’ formed for one of the stimulus-response pairings accessible in Stage 3. Top: As a result of having learned E2->R2 (Stage 1) and S4->E2 (Stage 2) associations, by transitivity S4->R2. Bottom: Example stimuli, responses, and outcomes taken from [2] are shown where green boxed images indicate trial relevant S->E->R->O. Key: Trial *A, B, C, D, E, F, G, H, I, J* = arbitrary trials; S1, S2, S3, S4, S5, S6 = Stimulus 1, Stimulus 2, Stimulus 3, Stimulus 4, Stimulus 5, Stimulus 6; R1, R2 = Response 1, Response 2; O1, O2 = Outcome 1, Outcome 2; E1, E2 = Outcome expectation 1, Outcome expectation 2; ∅ = no reward / incorrect response feedback.

While standard DOT entails a single stage of training (highlighted by the grey rectangle in Fig. 2 leftmost tabulated column), in contrast, Transfer of Control (ToC) procedures use DOT as one stage among several stages of training/testing (Fig. 2 table). ToC procedures have been used to provide evidence for the existence of a memory process governing response selection that encodes both an instrumental (stimulus-response) and a pavlovian (stimulus-outcome expectation) component. This so-called *Associative Two-Process theory* [6] posits that there exist two routes of stimulus ‘processing’ providing information for response selection. Firstly, there is a habit-based stimulus-response (S-R) route. Secondly, there is a stimulus-(outcome) expectation-response (S-E-R) route. These two routes are learned through associating their respective components (S-R and S-E plus E-R, respectively). The two routes are hypothesized to converge permitting outcome expectancy (E) to assert stimulus control to alternately *facilitate* [1], *substitute for* [7], or *override* [8,9] the influence of the discriminate stimulus (S), depending on the particular ToC methodology used. To test the existence of this Associative Two-Process, the ToC procedure requires adding a second stage of learning (Stage 2, Fig. 2 top left) to the standard one-stage DOT (Stage 1, Fig. 2 leftmost). In the case of Fig. 2 (left) a pavlovian stage serves for Stage 2 and entails passive learning of associations between novel stimuli and the differential outcomes that were accessible in Stage 1. In a test stage (Stage 3, Fig. 2 top left), participants undergo another instrumental stage similar to Stage 1 where the same response options are accessible but participants are now presented with the novel stimuli of Stage 2. The prediction, and typical finding, at this stage is that the participant will tend to have a selection bias towards one of the two responses in the first trials of this stage in spite of never having been presented the particular stimuli-response pairings in the previous stages of learning. By way of explanation, Fig. 2 left (top portion) shows the S-R-O contingencies of each of the three stages of the procedure, and (bottom portion) the Associative Two-Process predictions that are made regarding participants’ response selection tendencies. By Associative Two-Process theory participants have learned S-E and E-R associations. Through this learning the S-E-R (outcome expectancy) route can *substitute for* the lack of formation of the S-R (habit-based) route at the beginning of Stage 3. This can also be viewed as a type of associative transitive inference, i.e. where the participant has learned the relation between S and E and between E and R they have also *indirectly* learned the relation between S and R (in the example, S4->E2, E2->R2, so transitively S4->R2).

This is illustrated schematically in Fig. 2 (right, top) with respect to a single trial (S4 presentation) and with a practical example (for stimuli, response and outcomes) Fig. 2 (right, bottom).

The above-mentioned transitivity can occur in relation to not only pavlovian manipulations in Stage 2 but also Instrumental manipulations. Urcuioli [2], in his description of conditions under which stimulus classes may form, for example, refers to scenarios in which a second stage of learning utilizes novel S-R pairings whilst utilizing outcomes presented in Stage 1. So now S3->R3->O1 and S4->R4->O2, respectively. In the test stage when S1 is re-presented along with R3, R4 options, by transitivity, S1->E1 (Stage 1) and E1->R3 entails S1->R3 (equivalent for other S-R test pairings), which is the transfer of control found in subjects. A related phenomenon Stimulus Equivalence Theory [10] see also [11,12] concerns discriminative response selection according to *reflexivity, symmetry* and *transitivity*. Associative Two-Process theory would require extension to accommodate the first two components to address equivalence. For example, where response presentations may trigger outcome expectations or/and stimulus expectations and vice-versa (symmetry).

**Fig. 3:**
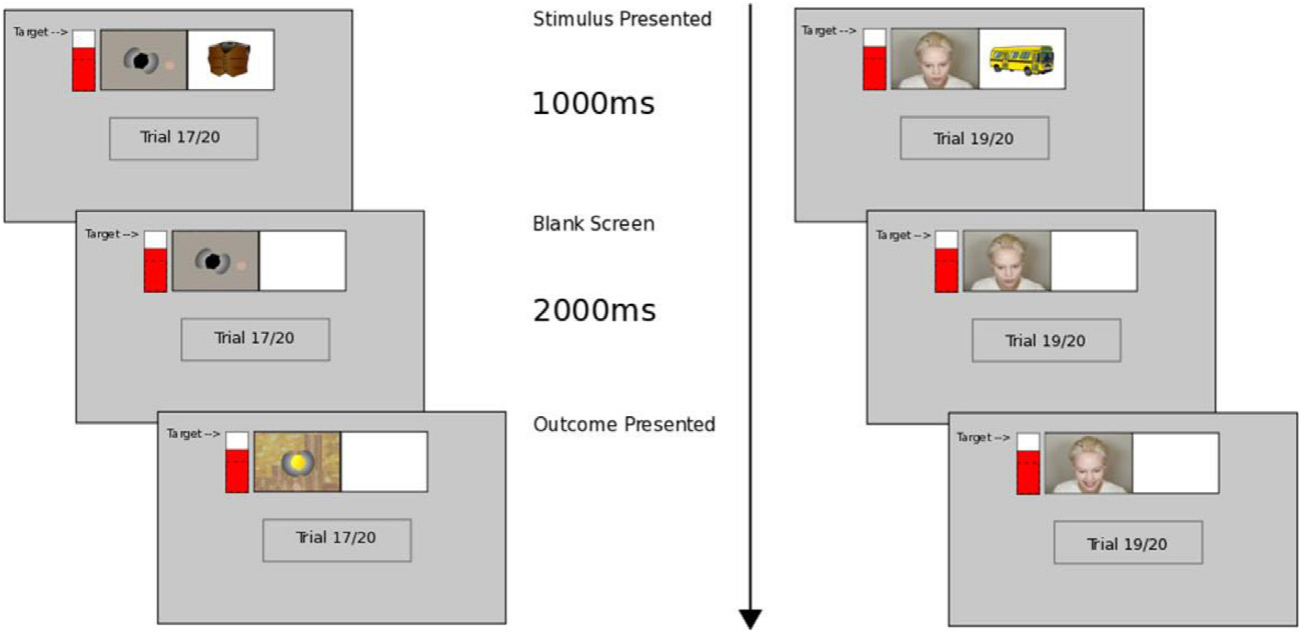
Example Trial Progression for Stage 2 of the Social ToC Experiment. Left. Non-social animated stimulus. Right. Social video stimulus (confederate). For each trial the participant was presented with a stimulus superimposed on the right white panel (top left/right) for 1000 ms, followed by a blank/white (stimulus) panel for a further 2000 ms. The video sequence in both social and non-social (control) conditions endured for the whole trial (left panel). All but the first trial was expressed as reward – the punishment/negative reward (shocked expression), was intended to increase believability in the confederate. In the non-social condition rewarding (monetary) outcomes were made explicit (faded in, see left bottom panel). The animation video in this non-social condition was used to control for the fact that the social condition used a video. The red filled score bar (left side of screen) displaying accumulated score remained at the level at which the participant had reached in Stage 1 (from [4]).

In [4] we provided an adaptation of the ToC protocol described above to a social context wherein lies our novel methodological contribution. The difference between the protocols concerns the implementation of Stage 2. Similar to the standard ToC the participant is required (and instructed) only to observe and learn rather than produce instrumental responses. However, this pavlovian component now involves the participant *observing* another (confederate) performing the task (with response options non-visible). The trial progression is visualized in Fig. 3, which shows both experimental (right), i.e. the social setting, and control (left) conditions for the particular implementation of this methodology in [4]. Here, a video stimulus in the social condition substituted for explicit outcomes (see Fig. 1 panel 4 left/right) as used in standard (non-social) ToC. The expression of the confederate provided the only cue as to the outcomes (high reward/low reward) through valenced facial expressions. This, therefore, represents a partially observable problem whereby neither response nor outcome are visible to the confederate. An alternative (also evaluated in [4]) concerns ensuring only response, but not outcome, is unobservable.

In [13,14] (see also [4,15]), we outlined a hypothesis as to the conditions under which so-called *vicarious value learning* could occur. Participants learn outcome expectations (value learning) in relation to Stages 1 and 2 of the ToC procedure. This learning may be vicarious in the sense of participants being able to learn the outcome expectancies of others through *placing themselves in the shoes of the other*. Mechanistically, this would require utilizing a single value function that is used for *self* when learning differentially rewarding outcomes for the value learning made by the observed *other* (confederate). This could occur through either a) cognitive empathy – mentally putting themselves in the shoes of the other, b) emotional empathy – through emotional contagion, feeling what the other feels. In the case of emotional empathy, it is assumed here that an emotional stimulus may automatically trigger emotional contagion tapping into empathic and value systems (see [16]). In a) and b) participants could directly tap into the value function in Stage 2 used for learning Stage 1 and therefore bring to bear Stage 1 and Stage 2 knowledge onto Stage 3 leading to the same theoretical predictions by Associative Two-Process theory as for the non-social ToC (Fig. 2).

Adapting the pavlovian transfer of control manipulation (Stage 2) to a social context is not without precedent. For example, [17] provide a review of the role of social pavlovian conditioning in shaping moral choice. They refer to vicarious learning whereby one subject can learn CS-US associations through observing others react to the CS. In the moral choice example given the CS is another human (‘receiver’). Social pavlovian conditioning has also been studied in relation to acquired fear through observing others being administered painful shocks [18]. Our social pavlovian conditioning by comparison concerns observation of others inducing positive reward value based associations (emotional contagion) through which associative transitive inference can be made.

## Transfer of Control: Procedural Methodological Considerations

In this sub-section we consider *procedural* methodological considerations for designing a human-participant transfer of control experiment. We consider three key components for creating the appropriate procedure for a transfer of control scenario using human participants: *Cognitive Load, Engagement*, use of *Quick-Fire Trials for Associative Learning*.

*Cognitive Load*: In order for a transfer of control to occur at all, participants must have been sufficiently well trained on the two stages that precede the test stage. Insufficient training could result in a lack of formation of the necessary E-R and S-E associations for the associative transitive bridge to be in place at the start of test stage (see Fig. 2, Right). This is easier to control for in the instrumental Stage 1 (e.g. through piloting). Low cognitive load [19] for the participants can be manipulated and evaluated in Stage 1 – this might manifest in few stimuli-response associations to be learned and be evaluated in terms of criterion performance (e.g. participants should achieve a high proportion of correct answers in the final blocks of the stage to demonstrate transferable learning). To implement this low cognitive load, participants were required to learn only two stimulus-response associations. A potential problem, however, in having just two associations to learn is that in a test stage, participants could score optimally through correctly guessing the response in the first trial, and then through process of elimination selecting the correct response when the alternative stimulus is presented on a subsequent trial. Obtaining performance differences between conditions can thereby be masked by such a non-targeted cognitive effect. To mitigate this, more stimuli can be included (process of elimination will be more difficult) with a resultant increase in cognitive load (we chose four novel stimuli for Stage 2).

*Engagement*: owing to the repetitive nature of the task (different stimuli are presented multiple times over alternate trials), If the cognitive load is too low, participants may *disengage* from the task [20] and subsequently perform sub-optimally. Disengagement, as a result of low cognitive load, may result in greater inter-participant cognitive and behavioural variability [21]. To counter this potential effect in Stage 1, we included a (red filled) score bar (see Fig. 3), which provided a running total score for the participant. Participants were told before the experiment that they would receive an external reward (cinema ticket) for achieving the “Target” score. This mechanism was thereby considered to help motivate participants extrinsically to concentrate on producing best performance over the stage.

*Quick-Fire Trials for Associative Learning*: A further consideration concerns the speed of the trial progression, i.e. delay between stimulus onset and offset and between stimulus offset and outcome presentations following response option selection. In order to tap into associative learning mechanisms and maintain engagement, therefore, such presentations should be in the order of seconds (e.g. 2–3 s). Naturally, piloting is still necessary to account for the *type* of participant. In memory training studies there can be a big difference in performance among target groups, e.g. young infants, elderly or adult students [3].

To summarize the above, ToC procedural methodological considerations should account for:

- *Cognitive Load*: Stage 1 should be of low cognitive load (in order for the E-R associative learning necessary for transfer of control to occur);
- *Engagement*: Additional motivation, e.g. accumulated score visualization leading to some real-world reward (payment) should be included. This could be seen as a form of gamification [22];
- *Quick-Fire Trials for Associative Learning*: Stages should be *quickfire* in order to promote associative forms of learning rather than alternative, e.g. deliberative forms, of learning not under investigation. See Fig. 1 and Fig. 3 for example durations of stimuli, delay, response options, as used in [4].

Naturally, order effects for conditions and stimuli need to be made.

## Social Transfer of Control: Procedural Methodological Considerations

The Social Transfer of Control (ToC) protocol differs from the standard ToC, in relation to Stage 2 of training (commonly a pavlovian stage). In [4], Experiment 2, our choice of social stimulus concerned the use of a video of a confederate (actress). An advantage of using a confederate as the social ‘stimulus’ is that the level of expression can be controlled for and also the same expressions can be used on all participants so as to reduce inter-participant variability.

A social stimulus could contain visual or/and audio components. A visual component, e.g. video, is commonly preferred for conveying a sense of social presence within the task owing to research into the expressive and neurophysiological constituents of visually expressive social cues (e.g. [23]). In [4], for the confederate's emotional expression to provide usable information on differential outcomes it is necessary to address a trade-off between *detectable expression versus credible expression*.

**Fig. 4:**
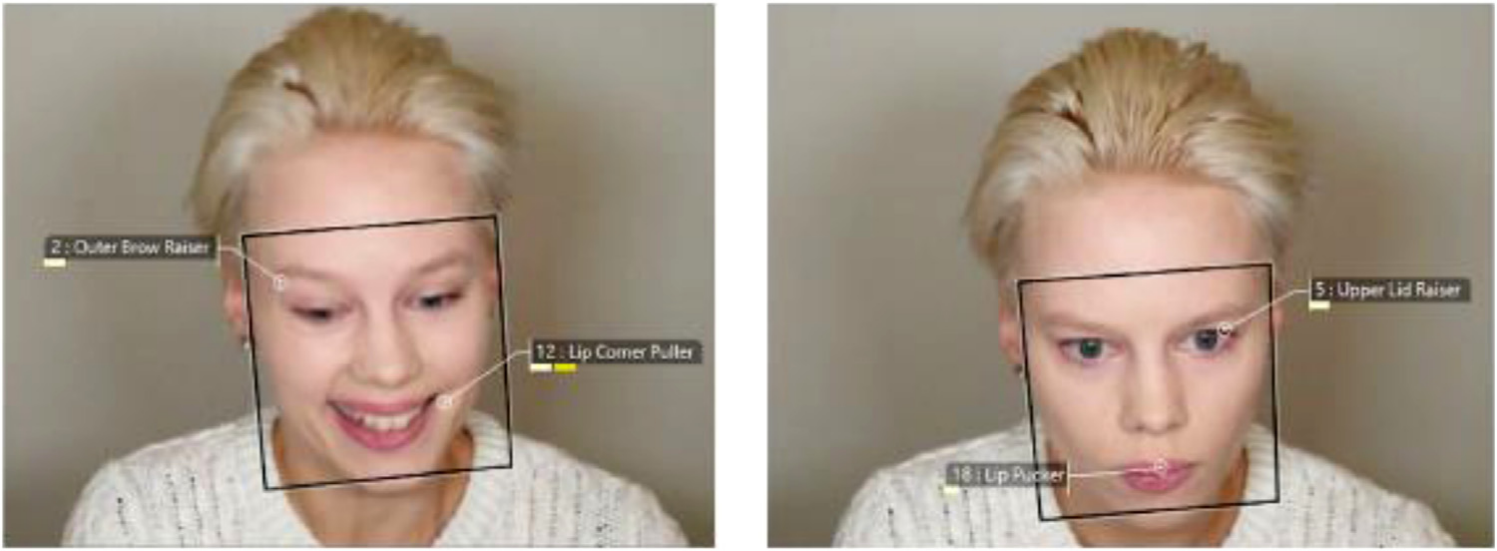
FaceReader facial expression analysis visualization of a confederate used in Stage 2 for the social condition(s). Left. The confederate expresses with positive valence. Right. The confederate expresses with negative valence. The outline box indicates the surface area over which FaceReader carries out the expression analysis. The numbered labels represent the Action Units that are expressed above baseline according to FaceReader (from [4]).

*Detectable Confederate Expression:* Use of emotion expression detection software can provide a reliable and scientifically grounded means to check the validity and reliability of a confederate's emotion expression in experiments. In pilot work for [4], a confederate was used and instructed to express naturally but in a somewhat animated manner to promote detectable emotion expressions. We provided an objective analysis of the validity of the facial expressions using Noldus FaceReader [24]. In Fig. 4 an example of the Action Units (AUs) picked up by FaceReader is shown. FaceReader was found to discriminate correctly between positive and negative valence of the facial expression in 19/20 video instances (i.e. one for each trial). In the only instance where the expression was picked up as negative valence when positive valence expression had been instructed to the actress (ground truth) it was the least negatively valenced expression. This small bias towards negative valence expression seemingly owes to the downward head positioning of the confederate that occasionally led to FaceReader interpreting a frown. Nevertheless, with re-calibration of valence threshold would have allowed all cases to be correctly marked as positive or negative. Note, FaceReader, and other emotion expression recognition software, provides instruction as to how to optimally set up lighting and camera position (in relation to head position) for optimal emotion expression detection. A conflicting variable (as for [4]) may be a task-specific requirement to sometimes look down (i.e. at the mouse during a computerized task) while the camera is typically mounted above screen. Potentially inter-rater coding could replace emotion recognition software for evaluating the quality of the confederate emotion expression^1^. Alternatively, this might be used in addition to emotion expression software at the cost of extra resources needed (time to carry out coding and criteria for coding). Ideally, multiple different confederate *social stimuli* would be used to control for any stimulus-specific effects on learning, e.g. gender.

**Fig. 5:**
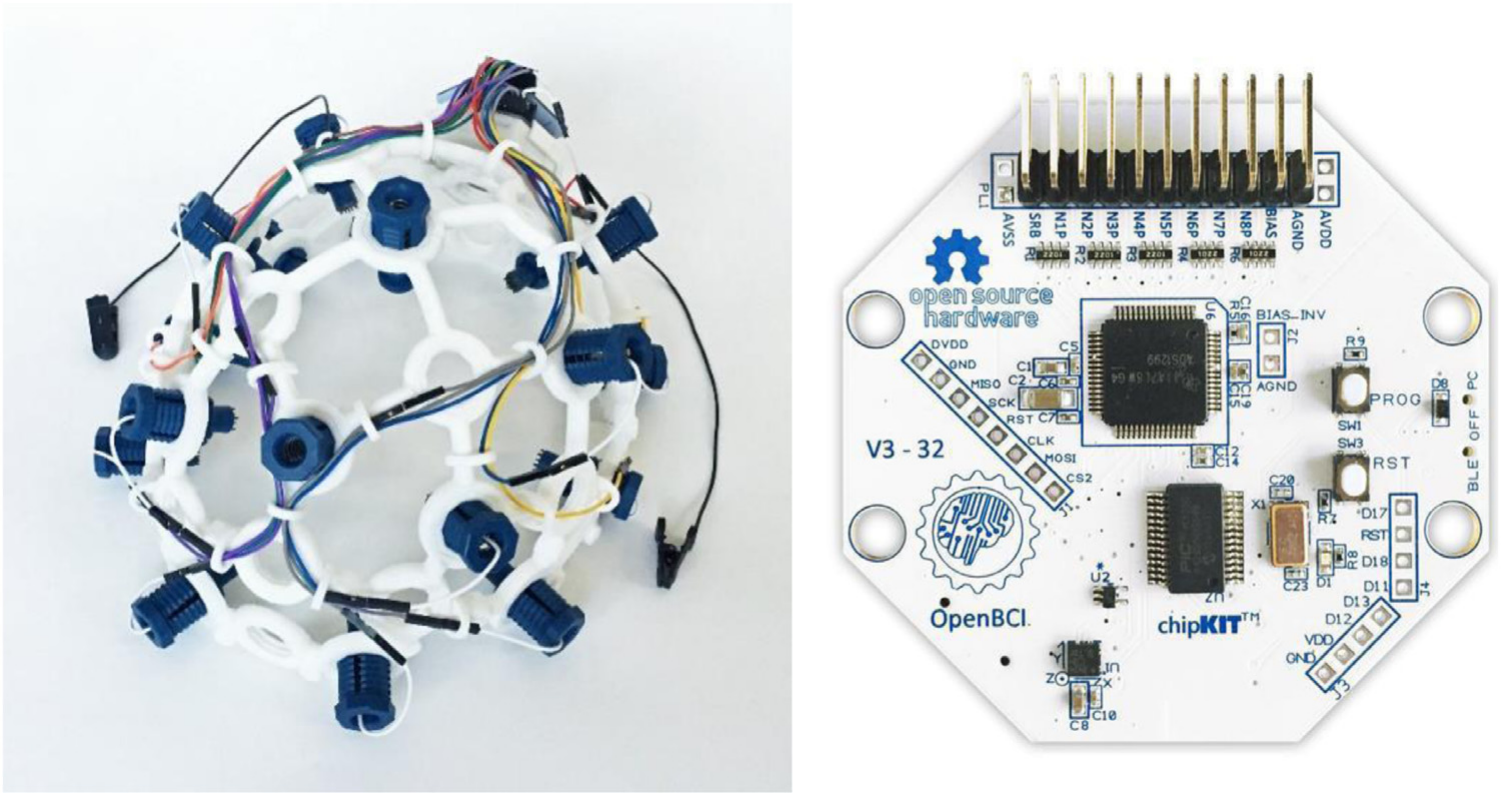
Portable and easy-to-set-up EEG equipment for Social ToC studies. Left. The OpenBCI-headset with 8 more electrodes than used in [4]. Right. The OpenBCI Cyton board.

*Credible Confederate Expression:* The credibility of the confederate's emotional expression is something that could potentially be gauged before a given experiment. Both objective and subjective measures can be evaluated. Note, in [4] we evaluated this after the experiment (Experiment 2) was carried out. Subjective measures can manifest in the form of questionnaires, interviews, etc., regarding how the participant felt about the confederate so as to gauge his/her credibility as an emotionally relevant social stimulus. Questions can be tailored to tease out cognitive and emotional empathic components, e.g. in relation to feeling ‘goals’ (cognitive) or feeling emotions. Objective measures, on the other hand, are required to be non-invasive so as to limit the impact on behavioural performance. In [4] we utilized a portable Electroencephalogram (EEG) headset, specifically the OpenBCI Cyton board. The Cyton board is an 8-channel neural interface, which samples data at 250 Hz. The associated OpenBCI headset Mark IV, based on the internationally recognized 10–20 system, was used and can be seen in Fig. 5. The headset is able to target 35 electrode locations of the 10–20 system.

Use of an easy-to-set-up EEG headset entails a trade-off between spatial resolution and invasiveness. The sum of neurons discharging creates the electric field and the farther away from the source of these discharges the activity is measured the harder it is to track any changes to a specific location. A further issue is the setup of the headset and a trade-off between *accuracy versus invasiveness*. This can be more challenging with more electrodes and if the procedure for placing the headset on the participant before the experiment begins is intricate it may detract from participants’ tendency to emotionally express in a natural way (for fear of displacing the electrodes on the scalp).

*Complexity of Non-Social Control Stimuli*: A non-trivial issue is providing a control for the ‘social stimulus’ (in [4], the social stimulus concerned the use of a video of a confederate expressing emotion in relation to differential rewarding outcomes; the control stimulus was an animated video within which differential rewarding outcomes were visible). The control stimulus must be: i) of the same type, e.g. video/animation; ii) of a comparable level of complexity. For video-based stimuli, since the confederate is expressive over the duration of each Stage 2 trial, the control stimulus must be similarly animated. In practice objective measures (EEG equipment) and subjective measures (questionnaire reporting) can be used after experimentation (or in a piloting phase) to assess the extent to which the experimental (social) stimulus involves a higher or lower cognitive load (when the participant is monitoring the animated stimuli).

To summarize the above, Social ToC procedural methodological considerations should account for:

- *Detectable Confederate Expression:* Emotional expression by the confederate is required to be detectable and repeatable over the trials in order for participants to assign the expression to differential outcomes. The expression, however, must not be stereotyped, i.e. it should be believable to the participants.
- *Believable Confederate Expression:* Objective and subjective measures are required to evaluate whether participants perceive the social stimuli as ‘social’ (and that the non-social stimulus is similarly non-social).
- *Complexity of Non-Social Control Stimuli*: Social and non-social control stimuli must be of a similar level of complexity such that associations with responses, outcomes and (*conditionable*) stimuli are not inherently more likely for one type of stimulus (social or control) or other.

Further considerations include having an unambiguous non-differential audio feedback signal for social conditions. This can allow for attention to be focused on the emotional expression at the time of the (unobservable) differential rewarding outcome.

## Validation

### Pre-conditions for Transfer of Control

There are two main aspects that need to be considered for validation of Social Transfer of Control experiments:

1) *Validation of the Transfer of Control (ToC)*:
  a. Stage 1 Validation: As discussed previously under *Cognitive Load*, it is needed to be addressed the question “Have participants learned sufficiently on this stage so that they have knowledge to ‘transfer’ to Stage 3?”
  b. Stage 2 Validation: A method for establishing whether failure to achieve ToC owes to lack of learning in Stage 2 or lack of ability to transfer learning to Stage 3.
2) *Validation of the Social Stimulus Applicability for Learning*:
  a. Detectable social stimulus expression validation: As discussed previously under Detectable Confederate Expression scientifically grounded software or expert inter-rater coder methods are required to evaluate the detectability of the emotions being expressed.
  b. Credible social stimulus expression validation: We advocate the use of objective and subjective measures as referred to in *Believable Confederate Expression*.

In order to address 1a), a criterion for “sufficient learning” must be set. Commonly, more or less arbitrary thresholds of performance (but above chance) are used as a metric for improved performance, typically in the final block of trials. For example, in [4] we used a threshold of 0.8 correct performance for the final block (of 5 trials) as a measure of learning. Similarly, in [19], a threshold of 0.75 was used to as a criterion for learning. Validation of 1b) is rather more challenging. Non-invasive EEG technology is typically too low resolution for it to provide reliable signals of learning (e.g. dopamine-based reward signals). Independent tests of pavlovian knowledge could be carried out, e.g. where questions are asked after a controlled test study to evaluate how well participants have learned as a means of calibrating the challenge level of Stage 2. While 2a. has been discussed in relation to validation in *Detectable Confederate Expression*, to address 2b if an EEG signal is to be used as a marker of social stimulus credibility, the electrodes must be placed in locations attributed to *social processing*. For example, C3 and C4 locations are considered candidates for relevant markers of social stimulus perception in that they are implicated in mirror neuron systems via suppression of mu rhythm activity.

For subjective feedback, questionnaires can be used to assess degree of empathy experienced with respect to the social stimulus and the type of empathy felt (cognitive versus emotional [16]).

Finally, computational modelling provides another means to put forward predictions regarding ToC performance, e.g. in terms of rate of learning and how the proposed two processes of Associative Two-Process theory neural dynamically converge to *facilitate, substitute for*, or *override* one another. Examples of such modelling can be seen in [25], [26], [27]. Such modelling can allow for testable hypotheses regarding the components of the model that provide necessary and sufficient explanations for the data. Modelling can also clarify understanding or bring to light issues that were imperfectly understood.

### Evaluating Transfer of Control

For assessing whether a transfer of control has occurred in the test Stage (3) of the transfer of control scenario (applicable to both social and individual variants of the paradigm), the following constraints are to be upheld:

a. Only an initial trial of blocks need be evaluated: Learning is not being assessed, rather direct transfer of knowledge from Stage 1 and Stage 2 onto the test Stage;
b. Differential outcomes condition performance (mean % correct on initial block of trials) should be greater than the control (non-differential outcomes or common outcomes conditions): For the control conditions, rewarding outcomes, through association with the novel stimuli of Stage 2 and the responses presented in Stage 1, should not provide an associative bias for one or other response option in Stage 3 unlike for the differential outcomes condition (see Fig. 2).
c. Differential outcomes condition performance (mean % correct on initial block of trials) should be greater than chance. Control condition performance need not be greater than chance.

Concerning the number of trials in the initial block, more than one trial is necessary to avoid excessive variance in the performance calculation. In [4] initial blocks consisted of 5 trials and so chance correct responding (given two response possibilities) was at 2.5 correct choices on average. The exact number of initial block trials may be arbitrary but should be at least as many as the number of novel stimuli introduced in Stage 2 so as to mitigate bias effects for particular stimuli. If stimuli presentation is random, there is a possibility that the same stimulus might be presented on multiple and successive trials. On this basis we selected more trials than number of new stimuli (used in Stage 2). Notwithstanding, we might expect some learning to occur in both conditions such that performance will be somewhat better than chance even for the control conditions (as was found in [4], Experiment 1 and 2 – see Figs. 5 and 9) – though it is possible to control for, or assess, number of stimuli repetitions in these blocks in order to infer a baseline (which in [2] amounted to control condition performance). An alternative would be to ensure that all N stimuli are presented (randomly) within the first N trials where N equals the number of new stimuli presented in Stage 2. However, this latter approach brings with it the possibility that participants can infer the correct response for the final 1 or 2 stimuli, e.g. if S3 and S5 are presented on trial 1 and trial 2 of Stage 3 and both require R1 to attain reward, the participant may infer that the stimuli presented on the subsequent two trials (S4, S6) require R2. Another possibility, not considered in [4] would be to compare Stage 3 performance on the first block of trials to Stage 1 performance on the first block of trials (of same size). In this case, we would expect a significant difference in performance on Stage 3 versus Stage 1 for the differential outcomes condition but not for the control (non-differential/outcome) condition. In [4] this comparison would not have been valid since only two stimuli were used in Stage 1 (so as to make relatively non-challenging – note, pre-conditions 1a for transfer of control above), as compared to four stimuli used in Stage 3 (and 2). As mentioned previously, the use of more stimuli in the test stage renders correct answers by a process of elimination, rather than associative inference, less likely. Allowing for four stimuli in Stage 1 with more trials in each of the learning stages could provide a potential solution at the risk of greater variance in performance on Stage 1. Mitigating this issue entails providing a second test stage whereby four novel stimuli are presented that have not previously been experienced. This would be the equivalent of Stage 1 matched for number of stimuli in Stage 3 and the stage would serve only as a means to evaluate the transfer of control. To control for order effects it would be necessary to randomize the order of presentation of the original Stage 3 and the additional test stage or otherwise have a between subjects design in relation to the final stage (the same subject may be prone to forget if the additional test stage occurs before the original test stage). Finally, the novel stimuli could be presented with response options where participants are then instructed that they can expect no feedback during this stage (to eliminate effects of learning). This might in turn compromise motivational effects (would participants continue to utilize the same outcome expectancy route?) but might provide a complementary form of control.

## Declaration of Competing Interest

The authors declare that they have no known competing financial interests or personal relationships that could have appeared to influence the work reported in this paper.

## Footnotes

1 In accordance with the FACS approach utilized by FaceReader, this would require coders being trained on Action Unit recognition (https://www.paulekman.com/facial-action-coding-system/).
